# Role of Multiparameter Analysis of AgNORs in FNA Smears of Thyroid Swellings in Differentiating Benign and Malignant Lesions

**DOI:** 10.1155/2012/908106

**Published:** 2012-06-24

**Authors:** Mohammad Ismail Hossain, Md Quamrul Hassan, Pradip Bhattacharjee, M. Shahab Uddin Ahamad, Zillur Rahman

**Affiliations:** ^1^Department of Pathology, Chittagong Medical College, Chittagong 4203, Bangladesh; ^2^Department of Pathology, Cox's Bazar Medical College, Cox's Bazar 4700, Bangladesh

## Abstract

*Background*. The aim of this study is to assess the role of multiparameter analysis of silver (Ag)-stained nucleolar organizer regions (AgNORs) technique on aspiration smears of thyroid swellings to distinguish between benign and malignant lesions. *Materials and Methods*. Aspiration smears from 166 cases of thyroid swellings were examined. Diagnosis was confirmed by histology in 61 cases. AgNOR staining was done on FNA smears according to silver-staining protocol proposed by the International Committee for AgNOR quantification. Multiparameter analysis of AgNORs such as mAgNOR, pAgNOR, and AgNOR size grade was done on 50–100 cells under oil immersion lens. *Results*. AgNOR parameter of benign and malignant thyroid lesions was compared and was found to be statistically significant. Out of 157 satisfactory AgNOR stained cases, 148 (94.3%) were benign lesions and 9 (5.7%) cases were malignant lesions. In AgNOR analysis, sensitivity was found to be 83.33%, specificity 100%, PPV 100%, NPV 98.21%, and accuracy was 98.36%. *Conclusions*. AgNOR analysis in the FNA smears is a simple, sensitive, and cost-effective method for differentiating benign from malignant thyroid swellings.

## 1. Introduction

The prevalence of thyroid nodules in the general population is about 5% by palpation and increases to 10–55% with the use of ultrasonography [1]. Thyroid nodules are common in women and in areas of iodine deficiency [2]. Benign thyroid disease is extremely common compared to small proportion of malignant neoplasm [1]. The distinction of the benign and malignant thyroid nodules is fundamental, as malignancy necessitates surgery [3]. To avoid unnecessary thyroid surgery, a thyroid scan, ultrasonography, and fine needle aspiration cytology (FNAC) are used as diagnostic tools to differentiate malignant from a benign lesion [4].

The major pitfall of FNAC is that it cannot differentiate between the follicular adenoma and follicular carcinoma [2]. This is why these are usually described as follicular neoplasm or thyroid follicular diseases [5]. The only diagnostic criteria of follicular neoplasm are the presence of vascular and capsular invasion that can be ascertained histologically [6].

Various attempts have been made to improve the diagnostic accuracy of FNAC, including morphometric study, DNA measurement, immunohistochemical and enzyme techniques for thyroid cancer with varying degree of success. It is well known that the silver staining technique for nucleolar organizer regions (AgNORs) has been successfully applied to a wide variety of neoplastic lesions on pathological materials in order to distinguish benign from malignant lesions [4].

The nucleolar organizer regions (NORs) are chromosomal loops of DNA involved in ribosomal synthesis [7]. In human karyotype, NORs are located on each of the short arm of the acrocentric chromosomes 13, 14, 15, 21, and 22 [8]. NORs are stained with silver nitrate (AgNO_3_) under proper conditions, and the structures visualized are termed as AgNORs. AgNORs can be identified as black dots in the nuclei [9]. The expression of AgNORs is associated with several biologic properties of cells: metabolic activity, DNA content, histological grade of differentiation, and, specially, the rapidity of cellular proliferation [10]. A high number of AgNOR dots are observed in proliferating cells [11]. Their size and number (mAgNOR) reflect nucleolar and cell proliferative activity of leisons [12]. The AgNOR proliferative index (pAgNOR index) is calculated according to procedure described by Mourad et al. as percentage of cells with 5 or more AgNOR dot [13]. Lesions having a pAgNOR count 8% or more were considered to display high proliferative activity [12].

In recent times, AgNOR analysis is carried through standardized morphometry. However, such techniques are not widely available specially in developing countries. The manual evaluation of AgNOR scores is a cost-effective alternative to automated methods of evaluation. The staining technique is relatively simple and rapid and can be applied to both aspiration smears and tissue sections [12]. The reliability of this method in cancer evaluation has been frequently demonstrated even by a simple visual assessment. Thus, counting of AgNOR dots appears to be very useful and simple way of obtaining data on the proliferative index of cancerous as well as benign lesions [14].

 The AgNOR count in malignant cells is significantly higher than that in normal and reactive cells. AgNOR size and dispersion are a more reliable and reproducible alternative to traditional AgNOR counts for differentiating benign and malignant lesions [15]. AgNOR size and dispersion are of higher grade in a significantly greater proportion of malignancy when compared with benign conditions [12]. Their qualitative profile, based on their size, shape, and dispersion, acts as a marker of premalignant/malignant change [16].

Hence, the aim of the study was to observe the role of multiparametric analysis of AgNORs, regarding the mAgNOR, pAgNOR, and AgNOR size rather than count (mAgNOR) alone as an adjunct to cytomorphological features in FNAC smears of thyroid lesions in differentiating benign and malignant lesions.

## 2. Materials and Methods

This prospective study was carried out in the Department of Pathology, Chittagong Medical College, Chittagong, Bangladesh, during the period from July 2010 to June 2011. A total of 166 patients with enlarged thyroid were selected consecutively for FNAC. From aspirated material, 4–6 smears were made, out of them 2–4 smears were immediately fixed in 95% ethyl alcohol for Papanicolaou stain and rest of the 2 smears were air dried and subsequently fixed in 95% alcohol for AgNOR stain. Cytological diagnosis was done from Pap-stained smear and multiparameter analysis of AgNORs done from AgNOR-stained smear to differentiate benign from malignant lesions. Utility of multiparameter analysis of AgNORs such as AgNOR count (mAgNOR), proliferative AgNOR (pAgNOR = percentage of cells containing 5 or more AgNOR dots), and AgNOR size grade (0–3+) was studied along with cytomorphological features, and results were confirmed by histopathology depending on the availability of specimens. Attempts were made to determine the diagnostic accuracy of conventional FNAC and AgNOR-stained smear in comparison to histopathological diagnosis.

AgNOR staining was done on FNA smears according to silver-staining protocol proposed by the International Committee for AgNOR quantification [17]. The argyrophilic proteins associated with nucleolar organizer regions (AgNORs) appeared as intranuclear black dots. Clusters of several dots were counted as a single unit if the dots could not be discerned separately. AgNOR-stained smears were examined under oil immersion lens in randomly selected 50–100 nuclei depending on availability of cells for following parameter.

### 2.1. AgNOR Count (mAgNOR)

The AgNOR score was expressed as mean AgNOR count (mAgNOR). mAgNORs were variable in different thyroid lesions, and there is overlapping between benign and malignant lesions, but usually more in malignant lesions. Less than 3 can be regarded as benign lesions according to findings of the study [4, 18].

### 2.2. Proliferative AgNOR (pAgNOR)

 AgNOR proliferative index (pAgNOR) = percentage of cells with 5 or more AgNOR dots [13, 19]. pAgNORs more than 8% were considered to display high proliferative activity. Malignant lesions show high proliferative activity. We consider <8 for benign lesions, 8–11 for suspicious lesions, and >11 for malignant lesions [13].

### 2.3. AgNOR Size Grade

The grading of size variation done according to method used by Khan et al. and scores of distribution are given in the following. AgNOR size grade is more in malignant lesions [6] (see Table 1): 

0: more or less uniform in size,

1+: two different sizes,

2+: more than two different size (but not those of 3+),

3+: including all size & grade.

### 2.4. Statistical Analysis

All the data was analyzed with statistical program for social sciences (SPSS version 18). The frequencies and percentages were presented for qualitative variables, and mean ± SD was presented for quantitative variables. All the relevant information was documented on a predesigned proforma. Statistical significance was evaluated at the *P* value =0.05 level. The sensitivity, specificity, positive predictive value, negative predictive value, and diagnostic accuracy of conventional FNAC and AgNOR study aspiration smears of thyroid lesions were calculated by using the histological diagnosis as gold standard.

## 3. Results

The patients were divided into six age groups. Age ranged from 15 to 70 years with maximum number 52 (31.4%) belonged to the age group 41–50 years. Mean age was 38.21 (SD ± 12.95) years. Male patients were 29 (17.5%), and female patients were 137 (82.5%) with a ratio of 1 : 4.71.

FNAC was done in 166 cases. On cytological examination, 24 (14.5%) were found to be malignant or suspicious lesions and 142 (85.5%) were benign lesions. Among the benign lesions, maximum 127 (76.6%) were of nodular goiter, 13 (7.8%) were of colloid goiter, and 2 (1.2%) were of thyroiditis. Among the malignant and suspicious lesions, maximum 19 (11.4%) were follicular neoplasm, 3 (1.8%) were papillary carcinoma, 1 (0.6%) was medullary carcinoma, and 1 (0.6%) was Hurthle cell neoplasm which is shown in Table 2.

**Figure 1:**
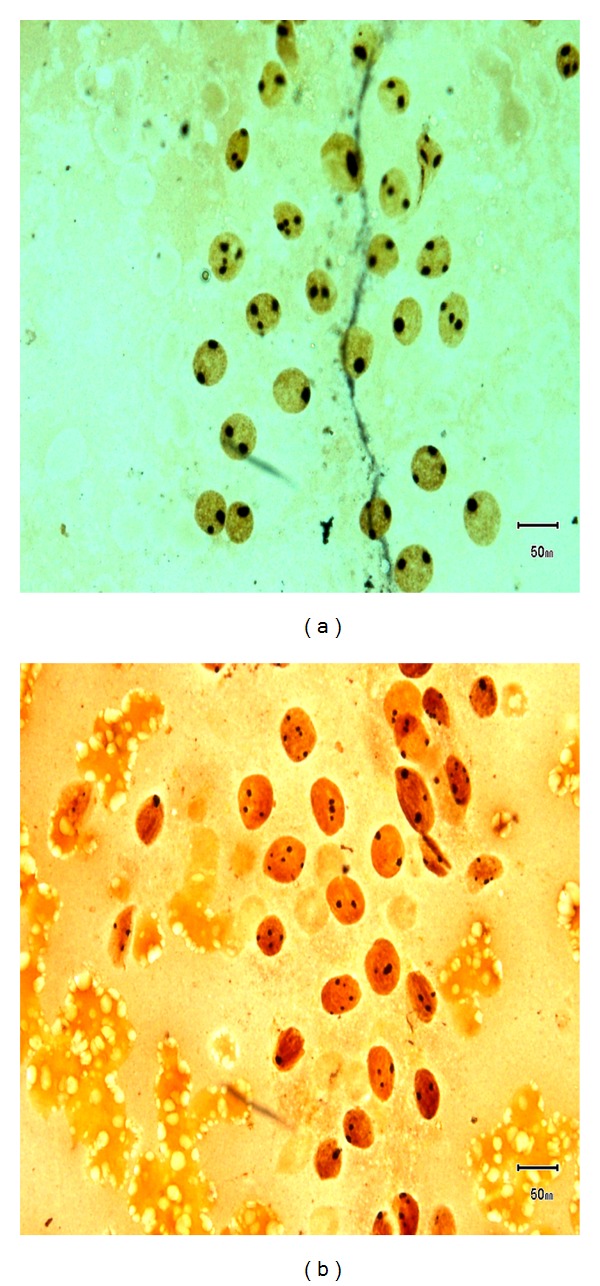
(a) AgNOR-stained smear of nodular goiter. (b) Papillary carcinoma (AgNOR stain) showing increased mAgNOR and pAgNOR.

**Figure 2:**
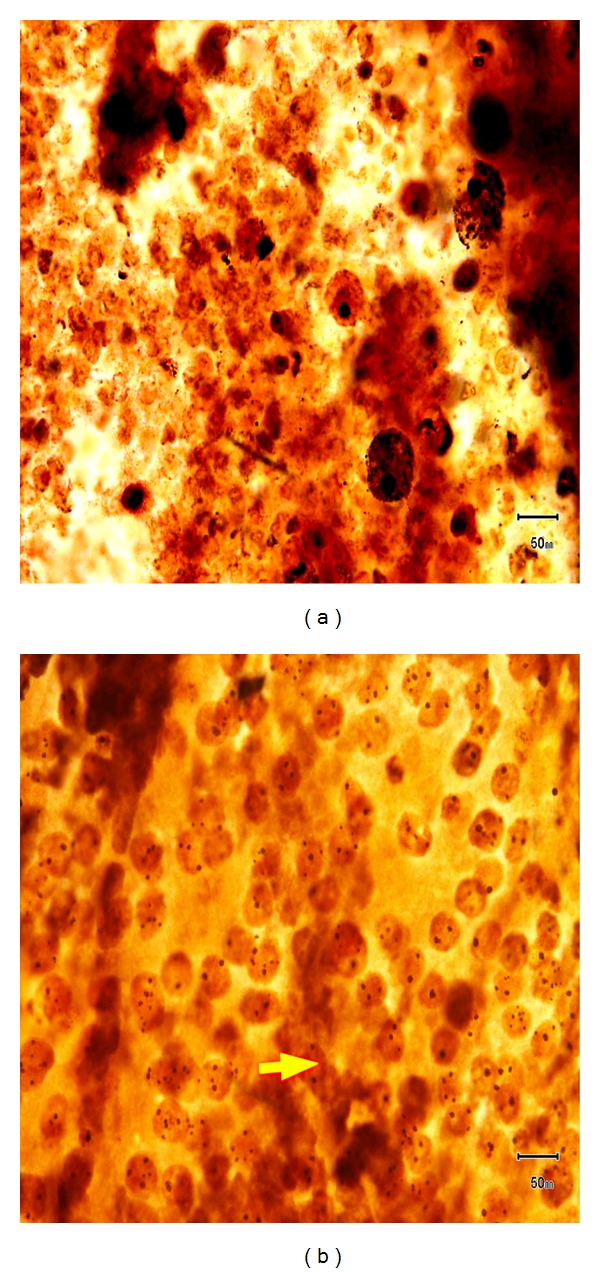
(a) Cystic lesions (AgNOR stain) showing macrophages. (b) Follicular carcinoma (AgNOR stain) showing increased pAgNOR.

**Figure 3:**
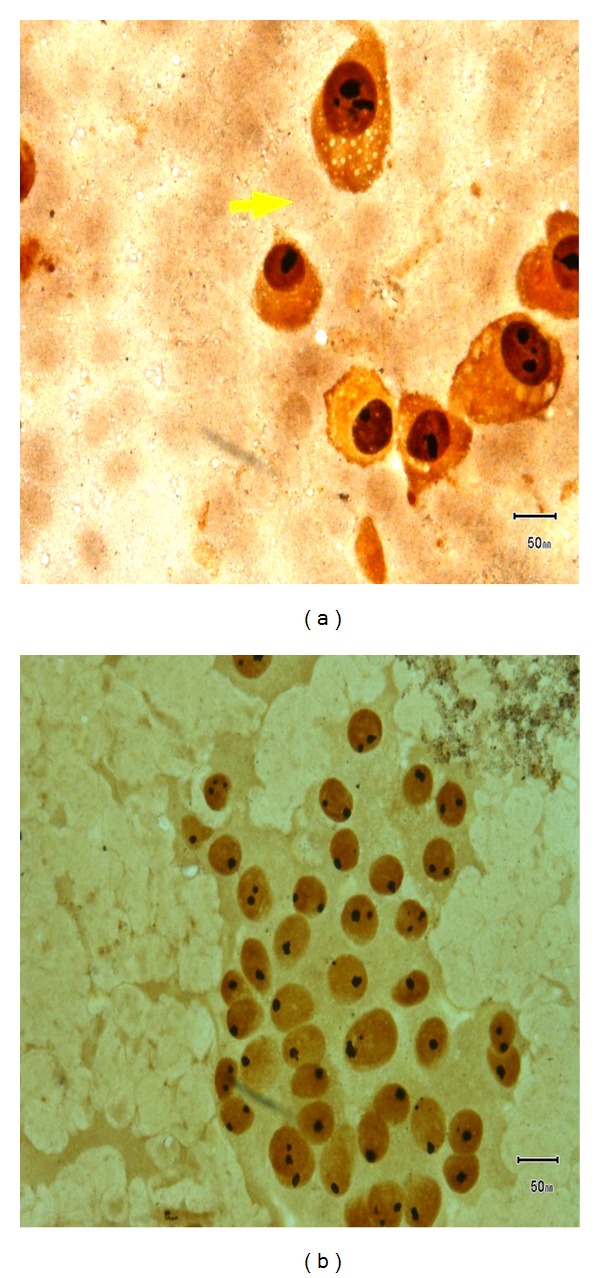
(a) Hurthle cell neoplasm (AgNOR stain). (b) Follicular adenoma (AgNOR stain).

AgNOR analysis was done on thyroid FNA smears. Out of 166 cases, 157 cases showed satisfactory smears for AgNOR analysis. Remaining 9 cases had unsatisfactory smears due to few or no cells with AgNOR stain. Out of 157 cases, 148 (94.3%) cases were benign lesions (Figures 1(a), 2(a), 3(a), and 3(b)) and 9 (5.7%) cases were malignant lesions (Figures 1(b) and 2(b)). Overall mean mAgNOR was 1.96 (SD ± 0.42) with a range of 1.14–3.30, mean pAgNOR was 1.39 (SD ± 3.45) with a range of 0.00–19.00. Mean mAgNOR in case of benign lesions was 1.91 (SD ± 0.36) with a range of 1.14–3.06 and in malignant lesions was 2.84 (SD ± 0.32) with a range of 2.49–3.30. Mean pAgNOR in benign lesions was 0.63 (SD ± 1.33) with a range of 0.00–7.40, and mean pAgNOR in malignant lesions was 14.02 (SD ± 3.01) with a range of 11.19–19.00. Grading of pAgNOR showed 148 had <8% pAgNOR and 9 showed >11% pAgNOR. AgNOR diagnosis showed 148 (94.3%) had benign lesions and 9 (5.7%) had malignant lesions. AgNOR size grading showed 45 (28.7%) 1+, 103 (65.6%) 2+, and 9 (5.7%) showed 3+ with much overlapping between benign and malignant lesions. All these findings are shown in Table 3.

Among the 166 cases of thyroid lesions, biopsy samples were available in 61 cases. Histopathological diagnosis showed 50 (82%) nodular goiter, 2 (3.3%) colloid goiter, 3 (4.9%) follicular adenoma, 5 (8.2%) papillary thyroid carcinoma, and 1 (1.6%) was medullary carcinoma. Overall benign lesions were 55 (90.2%), and malignant lesions were 6 (9.8%). The results are shown in Table 4.

FNAC results of 61 cases showed 51 cases with nodular goiter, 1 case with colloid goiter, 7 cases with follicular neoplasm, and 2 cases with papillary carcinomas. Histopathologically, out of 6 malignant cases, 4 FNAC reports are proved to be malignant, 2 as nodular goiter. Histopathologically out of 55 benign cases, 49 cases showed nodular goiter in FNAC report, 1 case with colloid goiter, and 5 cases showed follicular neoplasm. The association between FNAC and histopathologic diagnosis is shown in Table 5.

AgNOR diagnosis was available in all 61 histopathologically confirmed cases. The results showed benign diagnosis in 56 cases and malignant diagnosis in 5 cases. Out of 56 benign AgNOR diagnoses, 55 proved to be benign histologically and 1 case is malignant. Out of 5 malignant AgNOR diagnoses, all are proved to be malignant histologically. The association between AgNOR diagnosis and histopathologic diagnosis is shown in Table 6.

In fine-needle aspiration cytology (FNAC), the sensitivity was found to be 66.67%, specificity 90.91%, PPV 44.44%, NPV 96.15%, and accuracy was 88.52%.

In AgNOR study, sensitivity was found to be 83.33%, specificity 100%, PPV 100%, NPV 98.21%, and accuracy was 98.36%.

## 4. Discussion

Nodular thyroid disease is a common clinical problem. A vast majority of nodules are nonneoplastic. FNAC has certain limitations, which restrict its use as a simple diagnostic test, especially in thyroid neoplasm [4]. In present study, multiparameter analysis of AgNORs such as mAgNOR, pAgNOR, and AgNOR size grade along with cytomorphological features in FNAC smears of thyroid swellings was used to differentiate between benign and malignant lesions of thyroid.

Clinicopathologic characteristics of our study of all FNAC cases (*n* = 166) showed that females (82.5%) formed the major group with male to female ratio 1 : 4.71 and age ranged from 15 to 70 years with maximum of patients being 21–50 years of age. These findings are concordant with Asotra and Sharma [4]. In FNAC diagnosis, we found that 85% were benign, 11% were suspicious, and 4% were malignant. In histopathology (*n* = 61), we found that 90.2% were benign and 9.8% were malignant. These results are concordant with the findings of Gharib and Goellner [20] and Wahid et al. [21].

Solymosi et al. [22] had reviewed 51 cases of thyroid FNA smears with AgNOR analysis and found mAgNOR count in benign lesions 2.07 ± 0.58 and 2.40 ± 0.44 in malignant lesions with no cut-off value. Mehrotra et al. [14] reviewed 140 cases of thyroid smears with AgNOR analysis and found <3 mAgNOR count in benign lesions and >3 mAgNOR count in malignant lesions with no cut-off values. Asotra and Sharma [4] also found that there is no cut-off values for mAgNOR count in thyroid lesions. Our study showed that mean mAgNOR in case of benign lesions was 1.91 (SD ± 0.36) with a range of 1.14–3.06 and in malignant lesions was 2.84 (SD ± 0.32) with a range of 2.49–3.30 with overlapping values in both benign and malignant lesions. So we can suggest that mAgNOR count alone cannot differentiate benign and malignant lesions.

Slowinska-Klencka et al. [23], Solymosi et al. [22], and Bukhari et al. [15] suggest multiparameter analysis of AgNORs to differentiate benign from malignant lesions. We also use multiparameter analysis of AgNORs in FNAC smears of thyroid swellings as mAgNOR, pAgNOR, and size grade of AgNOR dots to differentiate benign and malignant thyroid lesions.

Our study showed that mean mAgNOR in case of benign lesions was 1.91 (SD ± 0.36) with a range of 1.14–3.06 and in malignant lesions was 2.84 (SD ± 0.32) with a range of 2.49–3.30. Mean pAgNOR in benign lesions was 0.63 (SD ± 1.33) with a range of 0.00–7.40, and mean pAgNOR in malignant lesions was 14.02 (SD ± 3.01) with a range of 11.19–19.00. Grading of pAgNOR showed 148 cases with <8% pAgNOR, and 9 cases showed >11% pAgNOR. AgNOR size grading showed that 45 (28.7%) case with 1+, 103 (65.6%) cases with 2+ and 9 (5.7%) cases showed 3+ with much overlapping between benign and malignant lesions. AgNOR impression showed 148 (94.3%) benign lesions and 9 (5.7%) malignant lesions.

Only 1 histopathologically proven case of papillary thyroid carcinoma showed <8 pAgNOR (false negative on AgNOR study), because only focal malignant changes seen in nodular goiter tissue and possibly smears could not be taken from malignant foci. In conventional cytology, there are 2 false negative cases of which 1 case later suggested malignancy on AgNOR stain. There was 5 false positive cases of follicular neoplasm in FNAC, which later suggested benign lesion on AgNOR study and subsequently proved as multinodular goiter or follicular adenoma histopathologically. Mean mAgNOR and pAgNOR values showed significant differences, specially pAgNOR revealed less overlap between benign and malignant thyroid lesions in histologically proven cases which is concordant with study done by Ansari et al. [12]. Our study showed that there are no significant differences in the AgNOR size grade between benign and malignant lesions which is discordant with Bukhari et al. [15].

Gharib and Goellner [20] had reviewed FNA smears of the thyroid, based on pooled data of seven series. The sensitivity and specificity were 65–98 and 72–100%, respectively. Our study showed sensitivity 66.67%, specificity 90.91%, PPV 44.44%, NPV 96.15%, and accuracy was 88.52% in conventional cytology. Comparison of results of present study with various studies is shown in Table 7.

AgNOR analysis showed sensitivity 83.33%, specificity 100%, PPV 100%, NPV 98.21%, and accuracy was 98.36% in differentiating benign from malignant thyroid lesions in FNA smears. Comparison of results of present study with various studies is shown in Table 8.

So, multiparameter analysis of AgNORs showed increased sensitivity and specificity than conventional cytology in FNA smears of thyroid lesions in differentiating benign and malignant ones.

One of the limitations of our study was lower biopsy sample for histopathological confirmation and also of unavailability of cases from every series, specially neoplastic cases. Another limitation was that there is no cut-off value for mAgNOR, AgNOR size grade. AgNOR size grading showed much overlapping between benign and malignant lesions. Mourad et al. [13] showed that pAgNOR > 8% indicates high proliferative activity and the malignant lesions showed high proliferative activity with a median of 11% for malignant lesions. It is a tedious procedure to manual analysis of 50–100 AgNOR stained cell, and there may be interobserver variation. On the other hand, computerized image analysis can estimate various parameters of the cell, but it is costly and unavailable to everywhere. A study with larger sample size may solve this type of limitations.

However, manual evaluation of AgNORs in FNA smears is very simple and less expensive as compared to other special methods like immunocytochemistry that are consistent with a series of studies [4, 7, 12, 14].

## 5. Conclusions

Multiparameter analysis of AgNORs in FNAC smears is a simple, sensitive, and cost-effective method for differentiating benign from malignant thyroid lesions and can be used as an additional diagnostic measure along with cytomorphological features.

## Figures and Tables

**Table 1:** Diagnostic criteria for AgNOR study.

Parameter	Benign	Suspicious	Malignant
mAgNOR	Variable, but usually <3	Variable, usually >3	Variable, usually >3
pAgNOR	<8%	8–11%	>11%
Size grade	0-1+	2+-3+, variable	2+-3+, variable

2 of the 3 parameters were taken to diagnose and confirmed by histopathology.

**Table 2:** Distribution of *FNAC findings* among the study subjects (*n* = 166).

FNAC diagnosis	Number of patients	Percentage (%)
Nodular goiter	127	76.6
Follicular neoplasm	19	11.4
Colloid goiter	13	7.8
Papillary thyroid carcinoma	3	1.8
Thyroiditis	2	1.2
Medullary carcinoma	1	0.6
Hurthle cell neoplasm	1	0.6

Total	166	100.0

**Table 3:** Distribution of *AgNOR diagnosis* (benign and malignant) (*n* = 157).

AgNOR diagnosis	Number of patients	Percentage (%)
Benign	148	94.3
Malignant	9	5.7

Total	157	100.0

**Table 4:** Distribution of *Histopathology findings* among the study subjects (*n* = 61).

Histopathological diagnosis	Number of patients	Percentage (%)
Multinodular goiter	50	82.0
Papillary thyroid carcinoma	5	8.2
Follicular adenoma	3	4.9
Colloid goiter	2	3.3
Medullary thyroid carcinoma	1	1.6

Total	61	100.0

**Table 5:** Correlation between FNAC and histopathology diagnosis (*n* = 61).

		Histopathology diagnosis		Total
		Benign (*n* = 55)	Malignant (*n* = 6)
FNAC diagnosis	Benign	50	2	52
	(*n* = 52)	(90.9) (96.2)	(33.3) (3.8)	(85.2) (100.0)
	Malignant	5	4	9
	(*n* = 09)	(9.1) (55.6)	(66.7) (44.4)	(14.8) (100.0)

Total		55	6	61
		(100.0) (90.2)	(100.0) (9.8)	(100.0) (100.0)

Figures in parentheses indicate percentages within columns and rows, respectively.

Chi-square test statistics: *χ* ^2^ = 14.259, *P* = 0.0002. Highly significant (*P* < 0.001).

Suspicious cases are considered as positive findings according to other authors [24, 25].

**Table 6:** Correlation between AgNOR and histopathology diagnosis (*n* = 61).

		Histopathology diagnosis		Total
		Benign (*n* = 55)	Malignant (*n* = 6)
AgNOR diagnosis	Benign	55	1	56
	(*n* = 56)	(100.0) (98.2)	(16.7) (1.8)	(91.8) (100.0)
	Malignant	0	5	5
	(*n* = 5)	(0.0) (0.0)	(83.3) (100.0)	(8.2) (100.0)

Total		55	6	61
		(100.0) (90.2)	(100.0) (9.8)	(100.0) (100.0)

Figures in parentheses indicate percentages within columns and rows, respectively.

Chi-square test statistics: *χ* ^2^ = 49.926, *P* = 0.0001. Highly Significant (*P* < 0.001).

**Table 7:** Comparison of FNAC results of present study with previous studies.

Study	Sensitivity	Specificity
Gharib and Goellner[20]	65–98%	72–100%
Khanzada et al. [26]	65%	98%
Lingegowda et al. [27]	66.5%	98.9%
Present study	66.67%	90.91%

**Table 8:** Comparison of AgNOR results of present study with previous studies.

Study	Sensitivity	Specificity
Chern et al. [28]	90%	90%
Palaoro et al. [29]	86%	100%
Present study	83.33%	100%
